# *Pm*_*SN15218*_: A Potential New Powdery Mildew Resistance Gene on Wheat Chromosome 2AL

**DOI:** 10.3389/fpls.2022.931778

**Published:** 2022-06-15

**Authors:** Meng Sun, Qi Liu, Yi Han, Guojun Liu, Jiajie Wu, Juan Qi, Fei Ni, Yinguang Bao

**Affiliations:** State Key Laboratory of Crop Biology, College of Agronomy, Shandong Agricultural University, Tai’an, China

**Keywords:** wheat, powdery mildew, BSR-Seq, *Pm4* loci, *Pm*_*SN15218*_

## Abstract

Powdery mildew, caused by *Blumeria graminis* f. sp. *tritici* (*Bgt*), is a devastating fungal disease that seriously damages the yield and quality of wheat in many regions of the world. Identifying new resistance genes and breeding new resistant varieties are effective methods to control this disease. The breeding line SN15218 shows good resistance against powdery mildew. We, therefore, developed an F_2_ population and 287 F_2:3_ families crossed between SN15218 and the powdery mildew susceptible cultivar Huixianhong (HXH). Genetic analysis indicated that a single dominant gene, designated herein *Pm*_*SN15218*_, conferred resistance to the *Bgt* isolate E09 in SN15218. Bulked segregant RNA-Seq (BSR-Seq) analysis revealed that *Pm*_*SN15218*_ is located in a ∼25-Mb interval on chromosome 2AL. Using the polymorphism information between SN15218 and HXH, we developed 13 polymerase chain reaction (PCR) markers and mapped this gene to a 0.5-cM genetic interval between the two flanking markers *PmM12* and *PmM14*, corresponding to a 6.01-Mb physical region in the Chinese Spring reference genome. The results of molecular marker analysis, allelic tests of resistance spectrum, and DNA resequencing indicated that *Pm*_*SN15218*_ is distinct from the known resistance gene *Pm4b* on 2AL.

## Introduction

Wheat (*Triticum aestivum* L.) is an important food crop worldwide, providing about 20% of human calories (Ni et al., 2017). However, wheat quality and yield stability are frequently affected by fungal diseases. For example, powdery mildew caused by *Blumeria graminis* f. sp. *tritici* (*Bgt*), is a devastating wheat foliar disease. In recent decades, powdery mildew has led to severe yield losses of 5–8% during the years of average infection, and as high as 40% in some years, in China and other parts of the world (Singh et al., 2016). In addition to fungicides and other biological agents, the deployment of disease-resistant cultivars is the most economical and environmentally friendly method for managing this foliar disease (Fu et al., 2013; Qie et al., 2019). However, because frequent changes in pathogen populations often overcome the effects of available resistance genes (Yu et al., 2018), most reported powdery mildew resistance genes in wheat are race-specific and susceptible to resistance loss once they are widely deployed in commonly planted cultivars. Therefore, it is necessary for breeders to discover additional sources of powdery mildew resistance genes in breeding programs.

At present, more than 100 powdery mildew-resistance genes or alleles mapping to 63 different loci (*Pm1*–*Pm68*, *Pm18*/*Pm1c*, *Pm22*/*Pm1e*, *Pm23*/*Pm4c*, *Pm31*/*Pm21*, *Pm48*/*Pm46*, and *Pm17*/*Pm8*) have been reported, with the loci *Pm1*, *Pm3*, *Pm4*, *Pm5*, and *Pm24* having 5, 17, 8, 5, and 2 alleles, respectively (Li et al., 2020; McIntosh et al., 2020; He et al., 2021; Zhang et al., 2021; Wu et al., 2022). These include five resistance alleles, *Pm4a*–*Pm4e*, reported at the locus *Pm4*, located on the long arm of chromosome 2A. *Pm4a* was first identified in the emmer wheat cultivar Khapli and the durum wheat cultivar Yuma and was linked to marker *Xgwm356* (Ma et al., 2004). *Pm4b* was derived from the French common wheat cultivar VPM1, a lineage derived from a cross between *Aegilops ventricosa*, *Triticum turgidum* var. *carthlicum* (*Triticum persicum*), and the common wheat variety Marne (Bariana and McIntosh, 1994), and has been mapped to a 3.0-cM genetic interval between flanking markers *Xics43* and *Xics13* in chromosome 2AL (Wu et al., 2018). Over time, wheat cultivars carrying *Pm4a* have gradually lost resistance to powdery mildew in many countries and regions, such as the United States, the Middle East, and China, while *Pm4b*, which was identified over 30 years ago, still provides effective resistance in parts of China and the United States (El-Shamy et al., 2016; Cowger et al., 2017; Wu et al., 2018). *Pm4c* (also called *Pm23*) comes from the common wheat line 81-7241, which exhibits high resistance to powdery mildew and has a broad resistance spectrum. *Pm4c* is located at a 4.9-cM genetic interval between *Xbarc122* and *Xgwm356* on chromosome 2AL (Hao et al., 2008). *Pm4d* and *Pm4e* were identified from the common wheat introgression line Tm27d2, which inherited its resistance from the *Triticum monococcum* accession Tm27 and the Chinese landrace D29, respectively. *Pm4d* was mapped to a 6.7-cM genetic interval, while *Pm4e* was precisely mapped to a 0.19-cM interval corresponding to a 360-kb physical region (Schmolke et al., 2012; Ullah et al., 2018). Recently, researchers successfully cloned the *Pm4a-Fed* and *Pm4b-Fed* genes by sequencing related mutants from the wheat lines Fed-Pm4a and Fed-Pm4b (Fed refers to the common wheat cultivar “Federation”), respectively, and identified multiple resistant or susceptible haplotypes of *Pm4-Fed* simultaneously (Sánchez-Martín et al., 2021).

Novel powdery mildew resistance genes identified from modern cultivars are easier to use in breeding than those from wheat relatives or wild species. However, the powdery mildew resistance genes so far identified are almost from landraces or wild relatives and often have adverse factors or linkage drag of undesirable genes. Only a few, such as *Pm2a*, *Pm4a*, *Pm6*, *Pm8*, and *Pm21*, originated from modern cultivars and can be directly used in breeding programs (Qie et al., 2019). Moreover, of the 18 resistance genes (*Pm51*–*Pm68*) newly identified in recent years, only *Pm52*, *Pm54*, and *Pm65* originated from adapted cultivars (Liangxing99, Pioneer 26R61, and Xinmai208, respectively) (Zhao et al., 2013; Hao et al., 2015; Li et al., 2019; He et al., 2021; Zhang et al., 2021).

SN15218 is derived from a natural mutant plant of the cultivar Yannong 19 (YN19) in the seed production field. It shows a high level of resistance to powdery mildew at both seedling and adult stages, suggesting that it harbors a powdery mildew resistance gene that could be valuable for wheat breeding. In this study, we characterized the resistance gene in SN15218, temporarily named *Pm*_*SN15218*_, through genetic analysis, molecular mapping, spectrum analysis, and allelic relationship comparison.

## Materials and Methods

### Plant Materials

The materials used in this study included the winter wheat cultivars YN19 and Huixianhong (HXH) and powdery mildew resistant line, SN15218. YN19 and HXH are widely planted commercial cultivars and are susceptible to most *Bgt* isolates. SN15218 is a breeding line with a YN19 background. It is similar to common wheat and shows resistance to many powdery mildew races. To analyze the inheritance of powdery mildew resistance, SN15218 was crossed with HXH to construct a segregating population, and F_1_, F_2_, and F_2:3_ materials were tested for resistance to the *Bgt* isolate E09, which is avirulent on SN15218 but virulent on HXH.

### Tests of Powdery Mildew Resistance

SN15218, HXH, and their F_1_ hybrids, F_2_ populations, and F_2:3_ families (25 seedlings for each line), as well as YN19 as a susceptible control, were tested for powdery mildew resistance using the *Bgt* isolate E09. Meanwhile, 23 single-spore-derived *Bgt* isolates and several wheat lines carrying known *Pm* genes were grown in a greenhouse at the Institute of Plant Protection, Chinese Academy of Agricultural Sciences, Beijing, to compare the reaction patterns of SN15218 and the other wheat lines with the different *Bgt* isolates. At the two-leaf stage, seedlings were inoculated with fresh spores and then transferred to a plant growth chamber at 15–18°C with 60% relative humidity and a 12 h light/12 h dark cycle to allow symptom development. Two weeks after the susceptible control materials HXH and YN19 were heavily infected, the plants were scored into infection types (ITs) according to a 0–4 scale (Tan et al., 2018). We classified the plants into two groups: resistant (R, IT = 0–2) and susceptible (S, IT = 3–4). Observed and expected segregation ratios were compared using the Chi-squared (χ^2^) test for goodness of fit.

### Bulked Segregant RNA-Seq Analysis

The F_2:3_ families with contrasting resistance phenotypes against isolate *Bgt* E09 (30 homozygous resistant and 30 susceptible families) were used to construct sample pools. Leaves of similar quality were collected from five plants of each family. Resistant and susceptible leaves were pooled for RNA isolation. Two parents, SN15218 and HXH, were also processed as parental checks. Total RNA was extracted using TRIzol reagent (Tiangen Biotech Co., Beijing, China) according to the manufacturer’s protocol. Library construction, high-throughput sequencing (Illumina NovaSeq 6000), and sequencing data qualification were performed by Berry Genomics Company (Beijing, China). Adapter sequences and low-quality bases were trimmed using fastp v0.19 with default paraments (Chen et al., 2018). High-quality reads were mapped to the Chinese Spring reference genome, RefSeq V1.0 (International Wheat Genome Sequencing Consortium, IWGSC), using Hisat2 v2.1.0 (Kim et al., 2019). Variant calling was performed using the HaplotypeCaller module in the GATK v3.8 toolkit (Poplin et al., 2018). Single-nucleotide polymorphism (SNP) filtration, sliding window analysis, and SNP-index plotting were conducted following a previously described protocol (Takagi et al., 2013). We excluded SNPs with read depth <5 from resistant and susceptible pools and two parent samples. SNP index values in the two pools were calculated using a custom perl script with SNPs of susceptible parent HXH as the reference, and the Δ(SNP-index) = (SNP-index of the resistant pool) − (SNP-index of the susceptible pool) was calculated for each SNP. Sliding window analysis was applied to ΔSNP-index plots with 1-Mb window size and 10-kb increment, and the average ΔSNP-index of SNPs was used for the sliding window plot (Takagi et al., 2013).

### Genotyping and Linkage Analysis

Leaf tissue was collected from 2-week-old plants, and total genomic DNA was extracted using the cetyltrimethylammonium bromide (CTAB) method (Porebski et al., 1997). According to the variants data from bulked segregant RNA-Seq (BSR-Seq) analysis, four polymerase chain reaction (PCR) markers were developed based on SNPs (Table 1). These markers were used to genotype the 285 F_2_ plants crossed with SN15218 and HXH and confirm the mapping result of BSR-Seq data. To develop more PCR markers, DNA resequencing and variants calling with two parents, SN15218 and HXH, were performed. The method of variant calling was similar to that described above for BSR-Seq, except that BWA v0.7.17^1^ was used instead of Hisat2 for mapping. Based on DNA resequencing data and the Chinese Spring RefSeq v1.0 reference map, seven insertion/deletion (InDel) markers were newly developed (Table 1) for further genotyping. χ^2^ tests of the F_2_ and F_2:3_ populations were used to evaluate the goodness of fit between observed data and expected segregation ratios. A genetic linkage map was constructed using the software JoinMap 4.0.^2^ The recombination values were converted to genetic distance (cM) using the Kosambi mapping function.

**TABLE 1:** Information of bulked segregant RNA-Seq (BSR-Seq) data.

Samples	Clean pairs	Clean bases (bp)	Read length (bp)	Mapping ratio (%)	Unique mapping ratio (%)	Note
Y2763	61,990,264	18,597,079,200	150	83.8	80.3	Resist parent SN15218
Y2764	62,628,016	18,788,404,800	150	83.6	79.9	Susceptible parent HXH
Y2765	63,695,851	19,108,755,300	150	85.9	81.9	Resistant pool
Y2766	58,922,100	17,676,630,000	150	84.0	80.1	Susceptible pool
Total	247,236,231	74,170,869,300	150	84.3	80.6	–

### *De novo* Assembly

DNA resequencing reads of the powdery mildew resistant line SN15218 were first aligned to the *Triticeae* repeat database mipsREdat 9.3p (PGSB Repeat Database) using BWA-mem v0.7.17 in order to filter out repeat noises. Non-mapped reads (which come from the genome region of non-repeat sequences) were fished using Samtools v1.9^3^ and then used for *de novo* assembly using SPAdes v3.13^4^ with default parameters.

## Results

### SN15218 Powdery Mildew Resistance Is a Monogenic Inherited Trait

**FIGURE 1:**
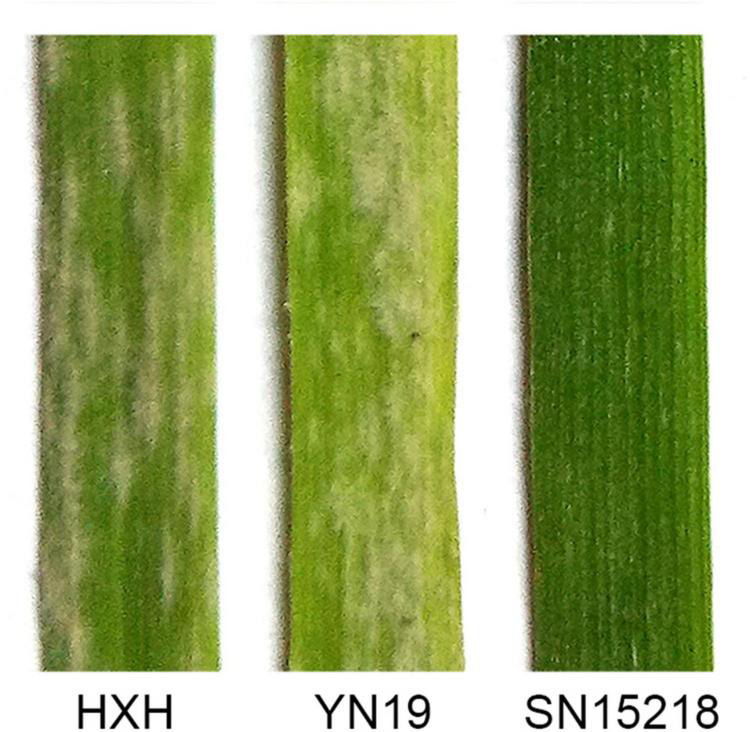
Powdery mildew infection of seedling from susceptible materials Huixianhong (HXH), Yannong 19 (YN19), and resistant parent SN15218 at 15 dpi inoculated with *Blumeria graminis* f. sp. *tritici* (*Bgt*) isolate E09.

When inoculated with *Bgt* isolate E09, SN15218 was highly resistant (IT = 0) and HXH was highly susceptible to the fungus (IT = 4) (Figure 1). F_1_ plants of SN15218 × HXH showed the same resistant phenotype as the parent SN15218, indicating the dominance of the powdery mildew resistance of SN15218. In the F_2_ population, 216 individuals showed resistance (IT = 0–2) and 69 individuals showed susceptibility (IT = 3–4), indicating segregation of a single resistance gene (χ^2^_3:1_ = 0.09, *P*_1df_ > 0.05). All the 285 F_2:3_ families derived from these two parents were also tested using the same *Bgt* isolate E09 in the climate chamber at the seedling stage. A total of 82 and 69 families were homozygous-resistant and homozygous-susceptible, respectively, and the remaining 134 families were segregated, confirming that the powdery mildew resistance of SN15218 was inherited as a single gene (χ^2^_1:2:1_ = 2.2, *P*_2df_ > 0.05). We temporarily named this resistance gene *Pm*_*SN15218*_.

### *Pm*_*SN15218*_ Gene Is Localized on Chromosome 2AL

**FIGURE 2:**
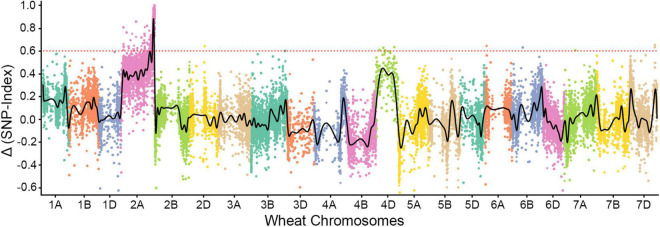
Bulked segregant RNA-Seq (BSR-Seq) analysis of *Pm_*SN*15218_*. *Pm_*SN*15218_* was localized at the distal end of the long arm of chromosome 2A.

We sequenced the two parents (SN15218 and HXH) and two resistant and susceptible descendant pools in 150-bp paired-end mode, generating 58.9–63.7 million clean paired reads; the average read depth was more than 60 for each SNP after filtration (Table 1). Approximately, 84.3% of the clean reads from each parent or pool could be mapped to the reference, and 80.6% of them were uniquely mapped and used for variant calling. We identified 46,895 high-quality homozygous SNPs between the two parents. Meanwhile, there were 1,027 and 1,791 homozygous SNPs in the resistant and susceptible pools, respectively, most of which were located on chromosome 2A (255 SNPs in the resistant pool, 645 SNPs in the susceptible pool). Only a single sharp peak (ΔSNP-index > 0.8) was identified on the long arm of wheat chromosome 2A, and these SNPs were enriched in a ∼25-Mb interval (Chr2A: Mb 751-776) (Figure 2), suggesting that the powdery mildew resistance gene of SN15218 was located on chromosome 2AL.

### *Pm*_*SN15218*_ Maps to a 0.5-cM Genetic Interval

**FIGURE 3:**
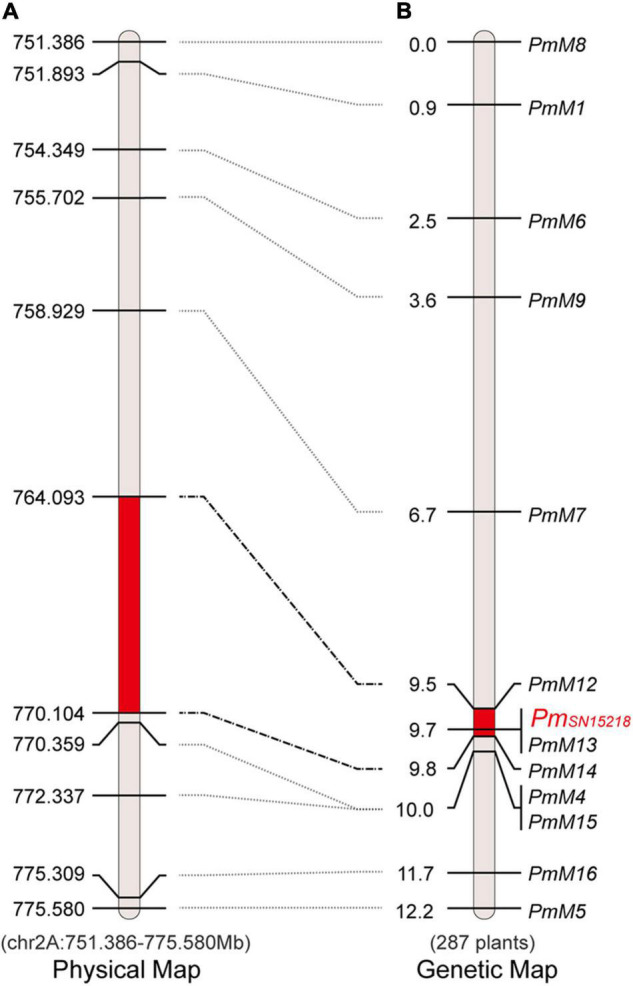
Physical and genetic map of *Pm_*SN*15218_* region. **(A)** Physical map of chromosome 2AL 751.4–775.6 Mb. **(B)** The precision genetic map with 287 F_2_ individuals. The map units were physical positions (Mb) or centimorgan (cM) that was labeled on the left sides of the map.

In the ∼25-Mb target region, we dispersedly selected five co-segregating SNPs/InDels identified from BSR-Seq data and converted them into cleaved amplified polymorphic sequences (CAPSs)/derived cleaved amplified polymorphic sequences (dCAPSs) markers (*PmM1*, *PmM4*, *PmM5*, and *PmM7*) or an InDel marker (*PmM6*) (Table 2). Using 90 F_2_ plants from the offspring of hybridization between SNP15218 and HXH, we genotyped these five markers and found that they were all closely linked to *Pm*_*SN15218*_, which confirmed the preliminary BSR-Seq mapping result. Based on DNA resequencing data, 6,172 SNPs/InDels were called in the ∼25-Mb target region, including 67 InDel sites larger than 10 bp between the 2 parents. We further created seven new InDel markers (Table 2) using the polymorphic information and reference data of Chinese Spring RefSeq v1.0 and mapped *Pm*_*SN15218*_ to the 0.5-cM *PmM12*–*PmM14* interval using 287 F_2_ plants, which corresponded to a 6.01-Mb physical region (chr2A: Mb 764.093–770.104) (Figure 3).

**TABLE 2:** Markers developed in this study.

Markers	Forward primer (5′ → 3′)	Reverse primer (5′ → 3′)	Tm (°)	Type^a^	Enz.^b^	Ploymorphic bands (bp)^c^
*PmM1*	GTGATGCTCGTATAACGCTTTCC	GTGGCTGCAATCATCAGCCTACC	60	CAPS	*Bsr*I	H (299); SN (248 + 51)
*PmM4*	TGCATGGAGTGCATCACCTAC	GATTTAGTTCTGTACTCATGAGTTG	60	CAPS	*Sty*I	H (233); SN (147 + 86)
*PmM5*	ACCCCAGCAAAACACGGAACAT	GCAAAAGGGGAATTGGGCCAT	60	CAPS	*Scr*FI	H (135 + 60); SN (195)
*PmM6*	TCATACTATGTGCTCAGCCAGA	AAGGAAGCGCAGCCTCGTGGT	58	InDel	–	H (234); SN (240)
*PmM7*	TGCAGAGGAAAATAGAGAATCAGGC	CATCGGTGGGTGCTGTTCCAT	54	dCSPS	*Nde*I	H (215); SN (193 + 22)
*PmM8*	GTGTAATTAGTGAGGTTGCTATAGC	CTACGCCGAAAGAATGTTCCTAC	58	InDel	–	H (371); SN (348)
*PmM9*	ACTCGGCGCACAGTCTGCTAT	GCCTCACGACATGGCTGGAT	58	InDel	–	H (178); SN (192)
*PmM12*	GGAGATACTCAAAGCAAAGCAAC	GTGCTACAAAGTGGGGAAATAATC	58	InDel	–	H (209); SN (226)
*PmM13*	GTCAGCTATCAGGACGAAATCGC	ACTCCTCAGTGCGTATTGCTG	58	InDel	–	H (185); SN (203)
*PmM14*	CCAAACCACAGCAACAGCCT	GGGATGATTAATTGGACGATCGA	58	InDel	–	H (155); SN (174)
*PmM15*	AAGATGGGCGCCGGGTAATG	GTCCTCAGAGCAAAATACTTCC	58	InDel	–	H (178); SN (199)
*PmM16*	GGGTATTCTGGTCATTTCTCGT	TAAGCGCCAGATAGGAGGC	58	InDel	–	H (203); SN (219)

*^a^Cleavage amplification polymorphism sequence (CAPS), degenerate cleavage amplification polymorphism sequence (dCAPS), insertion/deletion (InDel).*

*^b^Restriction enzymes (Enz.) used to digest the PCR product.*

*^c^Numbers within parentheses represent the size of diagnostic bands in HXH (H) and SN15218 (SN).*

### Resistance Spectra

In addition to *Pm*_*SN15218*_ from SN15218, several powdery mildew resistance genes, *Pm4a*, *Pm4b*, and *Pm4c*, that were previously identified from different wheat varieties or lines (Khapli, Armada, and 81-7241, respectively) were also mapped on chromosome 2AL. We evaluated the disease reactions of SN15218 and the above resistant landraces to 23 *Bgt* isolates. Line 81-7241, carrying *Pm4c*, showed high resistance to all of the isolates. Khapli, carrying *Pm4a*, showed resistance to most powdery mildew isolates but was susceptible to seven isolates. Armada, carrying *Pm4b*, showed a similar resistance spectrum to Khapli except for two isolates, E05 and E15 (Table 3). Line SN15218 exhibited better resistance than Khapli and Armada and was susceptible to only four *Bgt* isolates, E18, E20, E31, and E32 (Table 3). The results demonstrated that the resistance spectrum of *Pm*_*SN15218*_ was distinct from that of *Pm4a*, *Pm4b*, or *Pm4c*.

**TABLE 3:** Resistance spectra of *Pm*_*SN15218*_ and different *Pm4* genes.

Lines	Genes	*Bgt* isolates																						
		E01	E02	E05	E06	E07	E09	E11	E13	E15	E16	E17	E18	E20	E21	E23	E26	E30	E31	E32	E49	E50	E60	E69
Khapli	*Pm4a*	1 + 0;	0	0	0	4	0;	0	0	3	0	1	4	4	0;	0	0	3	4	4	0;	0	0	0
Armada	*Pm4b*	0;	0;	3 + 0;	0	4	0;	2 + 0;	0;	0;	0;	0;	3	3	0;	0;	0;	3	4	3	0	0	1 + 0;	0
81-7241	*Pm4c*	0;	0	0;	0;	0	0;	0	0	0;	0;	0;	0	1 + 0;	0;	0;	0;	0	0;	0	0;	0	0	0
SN15218	*Pm*_*SN15218*_	0;	0;	0;	0;	0	0	0	0;	0;	0;	0	4	4	0	0;	0	1	4	4	0;	0	0;	0;

*“0” and “0;” represent immune and near-immune, respectively.*

### Allelic Relationship

To compare the physical locations of *Pm* genes at the *Pm4* locus, including *Pm4a* (Hao et al., 2008; Fu et al., 2013), *Pm4b-Fed* (Sánchez-Martín et al., 2021), *Pm4c* (*Pm23*) (Hao et al., 2008), *Pm4d* (Schmolke et al., 2012), *Pm4e* (Ullah et al., 2018), *Pm65* (Li et al., 2019), and *Pm*_*SN15218*_, we integrated these tightly linked markers into chromosome 2AL of the Chinese Spring reference genome (IWGSC RefSeq v1.0) (Supplementary Table 1). These seven genes were located at the same physical interval: *Pm4a* was 4.8cM from marker *Xgwm365*, which was located in a region around Mb 762 of chromosome 2A; *Pm4b-Fed*, *Pm4c* (*Pm23*), *Pm4d*, *Pm4e*, *Pm65*, and *Pm*_*SN15218*_ were located in the physical interval of Mb 760–770. Among them, the *Pm4b-Fed* gene, originating from tetraploid *T. carthlicum*, is not in the “Chinese Spring” genome reference but is most closely related to the gene *TraesCS2A01G557900* near Mb 761 of chromosome 2AL, the ancestor of which was duplicated and fused to form the kinase structural domain of *Pm4b-Fed* (Sánchez-Martín et al., 2021). To further confirm the relationship between *Pm*_*SN15218*_ and *Pm4b-Fed*, we performed a PCR assay using three *Pm4*-specific PCR markers, *JS717* × *JS718*, *Pm4.1*, and *Pm4b-Fed-S* (newly developed in this study), and the results showed that SN15218 did not contain *Pm4*. We also performed *de novo* assembly using DNA resequencing data of SN15218 and found no *Pm4b-Fed* gene or fragment in the assembly database. In addition, we compared five other *Pm* genes, *PmX* (Fu et al., 2013), *PmLK906* (Niu et al., 2008), *PmPS5A* (Niu et al., 2010), *PmMl92145E8-9* (Yu et al., 2018), and *PmXMM* (Yao et al., 2022), which were also located on chromosome 2AL (Supplementary Table 1). Three of these, *PmX*, *PmLK906*, and *PmPS5A*, shared similar physical locations with *Pm*_*SN15218*_, but *PmMl92145E8-9* and *PmXMM* were located in the proximal region of the *Pm4* locus.

## Discussion

Powdery mildew is a widespread fungal disease of wheat, which can infect wheat at both the seedling stage and adult stage and causes significant yield losses. Loss of resistance in wheat cultivars caused by the rapid evolution of this pathogenic fungus has prompted a continuing search for new sources of resistance. Here, we identified a single dominant resistance gene (*Pm*_*SN15218*_) near the *Pm4* loci derived from the resistant mutant SN15218 through genetic analysis, molecular mapping, resistance spectrum investigation, and allelic relationship analysis. We tested different wheat genotypes against 23 *Bgt* isolates. The SN15218, carrying *Pm*_*SN15218*_, displayed a different resistance pattern from wheat carrying *Pm4a*, *Pm4b*, or *Pm4c*. SN15218 was highly resistant to powdery mildew in fields and has the genetic background of common wheat. Therefore, it could serve as a good intermediate material for powdery mildew resistance breeding.

**FIGURE 4:**
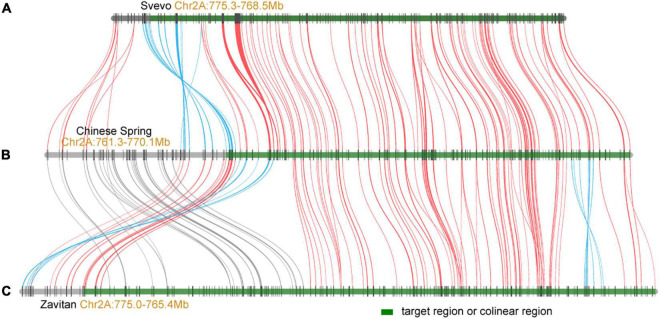
Collinearity of *Pm*_*SN15218*_ region between durum wheat Svevo **(A)**, common wheat Chinese Spring **(B)**, and wild emmer wheat Zavitan **(C)**. The green regions represent the colinear intervals of *Pm*_*SN15218*_ gene.

The *Pm*_*SN15218*_ was mapped to a 0.5-cM genetic interval flanked by markers *PmM12* and *PmM14*, responding to the physical region of Mb 764.1–770.1 on chromosome 2A in the Chinese Spring RefSeq V1.0. One hundred and twenty-three genes were annotated in this region. Among them, 12 encode for NBS-LRR-like resistance proteins or disease-resistance proteins, and 11 encode for receptor-like kinases or protein kinases. Comparative genomics analysis indicated that most genes in the *Pm*_*SN15218*_ region were conserved between the common wheat Chinese Spring (IWGSC), wild emmer wheat Zavitan (Avni et al., 2017), and durum wheat Seveo (Maccaferri et al., 2019; Figure 4). However, there were some inversions between Chinese Spring and Svevo or Zavitan and small chromosome fragment translocations between Chinese Spring and Zavitan in the upstream flanking region at the proximal side (Figure 4). These results indicate that the colinear genes located upstream of the target region should be considered when filtering candidate genes. In addition, more molecular markers and a larger segregation population need to be developed to enable further fine mapping and cloning of *Pm*_*SN15218*_.

The *Pm4* loci for powdery mildew resistance in wheat are complex, and much research has focused on its localization, cloning, and breeding applications. Previous studies have shown that *Pm4b* is localized in the physical interval of Mb 771.887–779.732 on chromosome 2A (Wu et al., 2018; Supplementary Table 1). However, Sánchez-Martín et al. (2021) cloned the *Pm4b-Fed* gene from the wheat cultivar Federation using the MutChromSeq approach, and their analysis revealed that this gene was localized near Mb 761 on chromosome 2A. *Pm4b* is derived from the French cultivar VPM1, and VPM1 drives from a cross between *A. ventricosa*, *T. turgidum* var. *carthlicum*, and the common wheat (*T. aestivum*) variety Marne (Bariana and McIntosh, 1994). It was introduced to China from Europe in the 1980s for genetic improvement of wheat disease resistance breeding. *Pm4b-Fed* is derived from three wheat lines, Weihenstephan M1, ELS, and TP229, and was first named *Mle*, which was identified as an allele of *Pm4a* and then transferred to the Australian spring wheat variety Federation by backcrossing (Briggle, 1966; Wolfe, 1967; McIntosh and Bennett, 1979). Since the two genes have different donors, the contradictory localization results could be due to ectopic recombination that occurred during transfer to the common wheat background. To determine the allelic relationship of *Pm_*SN*15218_* and *Pm4b-Fed*, we tested *JS717* × *JS718* and *Pm4.1*, two specific markers for *Pm4b-Fed* (Sánchez-Martín et al., 2021; Yao et al., 2022), and a newly developed marker, *Pm4b-Fed-S* (Supplementary Table 1), and found that this gene was not present in HXH, YN19, or SN15218. In addition, we analyzed the closely linked markers of other *Pm* genes, including *PmX*, *Pm65*, *PmLK906*, *PmPS5A*, and *PmMl92145E8-9*, on chromosome 2AL using *in silico* PCR (Supplementary Table 2). These markers are either undetectable or differ in product size in the *de novo* assembly database of SN15218. Combining the results of the genetic map, resistance spectra, allelic relationship, and DNA resequencing data, we speculate that *Pm_*SN*15218_* may be a potential new powdery mildew resistance gene, distinct from *Pm4*, on chromosome 2AL.

## Conclusion

Herein, we identified a wheat powdery mildew resistance gene, *Pm*_*SN15218*_, located on chromosome 2AL. It is distinct from the previously cloned *Pm4b*. This gene was derived from the breeding line SN15218 and could serve as a valuable genetic resource for wheat powdery mildew resistance breeding without any adverse factors or linkage drag of undesirable genes.

## Data Availability Statement

The original contributions presented in this study are included in the article/Supplementary Material, further inquiries can be directed to the corresponding authors.

## Author Contributions

YB, FN, and JW designed the project. MS, QL, YH, GL, and JQ performed the experiments. YB, FN, and JQ analyzed the data and wrote the manuscript. All authors discussed the results and reviewed the manuscript.

## Conflict of Interest

The authors declare that the research was conducted in the absence of any commercial or financial relationships that could be construed as a potential conflict of interest.

## Publisher’s Note

All claims expressed in this article are solely those of the authors and do not necessarily represent those of their affiliated organizations, or those of the publisher, the editors and the reviewers. Any product that may be evaluated in this article, or claim that may be made by its manufacturer, is not guaranteed or endorsed by the publisher.

## Footnotes

1
https://github.com/lh3/bwa

2
https://www.kyazma.nl

3
http://www.htslib.org

4
https://github.com/ablab/spades

## Supplementary Material

Supplementary Table 1 Closely linked markers for several reported powdery mildew resistance genes on chromosome 2AL.

Supplementary Table 2 *In silico* polymerase chain reaction (PCR) analysis of selected powdery mildew resistance genes on chromosome 2AL.
