# Supplementary material for: PmSN15218: A Potential New Powdery Mildew Resistance Gene on Wheat Chromosome 2AL
Source: Front Plant Sci. 2022 Jun 15;13:931778. doi: 10.3389/fpls.2022.931778 (PMC9240466; doi:10.3389/fpls.2022.931778)
Supplement: Supplementary Table 1 — Closely linked markers for several reported powdery mildew resistance genes on chromosome 2AL. [file Data_Sheet_1.docx]

Supplementary Material

# Supplementary Tables

Supplementary Table 1. Closely linked markers for several reported powdery mildew resistance genes on chromosome 2AL

| **Gene** | **Flanking marker** | **Forward primer sequence** | **Reverse primer sequence** | **Chromosome positions** | **Reference** |
| --- | --- | --- | --- | --- | --- |
| *Pm4a* | *Xgwm356-2A* | TCCAGTGACCCCATCTGCTCATAC | TCTCAGCGTTCCTTGTGATTCC | 762,462,195 - 762,469,567 | (Hao et al., 2008) |
|  | *XResPm4* | TGTCCGGTTTGGTTACCTTTCTTC | GGGACGCTTCCTATAATCACGC | ND^b^ | (Fu et al., 2013) |
| *Pm4b* | *Xics13* | AGGGAAATACTGACGTAGCTT | GTCAAGAGGAAGAAGGAAAAG | 771,886,798 - 771,887,049 | (Wu et al., 2018) |
|  | *Xics43* | CCCACCTGTCATACTCTGTT | CTCTGGCCCAATGATAGC | 779,731,943 - 779,732,143 |  |
| *Pm4c/Pm23* | *Xgwm356-2A* | TCCAGTGACCCCATCTGCTCATAC | TCTCAGCGTTCCTTGTGATTCC | 762,462,195 - 762,469,567 | (Hao et al., 2008) |
|  | *Xbarc122-2A* | CCCGTGTATATCCAGGAGTG | CAGCCCTTGTGATGTGATG | 766,164,161 - 766,164,429 |  |
| *Pm4d* | *Xgwm526-2A* | CAATAGTTCTGTGAGAGCTGCG | CCAACCCAAATACACATTCTCA | 760,575,627 - 760,575,776 | (Schmolke et al., 2012) |
|  | *Xbarc122-2A* | CCCGTGTATATCCAGGAGTG | CAGCCCTTGTGATGTGATG | 766,164,161 - 766,164,429 |  |
| *Pm4e* | *Xwgrc908* | CAAGGTTGATAAGTGTCTGTGGGC | GCGAAGTTCATTGGTCCGTTG | 761,900,111 - 761,900,334 | (Ullah et al., 2018) |
|  | *Xwgrc865* | TGAACTGTGAGGTATCCCACCC | GCTTCATCTTTCCCCCCTTC | 768,035,275 - 768,035,718 |  |
| *PmM192145E8-9* | *Xsdauk13* | GCTAAAGTGGACTACTAGAGGCAG | ATTTAGCTTTGTTCAATAGGTTCAC | 728,609,329 - 728,609,534 | (Yu et al., 2018) |
|  | *Xsdauk682* | GCGCATTTGAGCTTTTTGTT | CGCCAGAGAATGTTTGTTCA | 737,338,408 - 737,338,658 |  |
| *PmXMM* | *2AL15* | CGCAAGTTACTAAGATGGAAAAC(G) | CCTCGTGGAGATAGAGCAGC | 755,705,177 - 755,705,252 | (Yao et al., 2022) |
|  | *2AL34* | ACAACACCATCTGTAATGTACAAG(C) | CCCGTCCTTGCAGTGAAATC | 759,456,729 - 759,456,691 |  |
| *PmLK906* | *Xgdm93-2A* | AAAAGCTGCTGGAGCATACA | GGAGCATGGCTACATCCTTC | 760,575,587 - 760,575,721 | (Niu et al., 2008) |
|  | *Xgwm265* | TGTTGCGGATGGTCACTATT | GAGTACACATTTGGCCTCTGC | ND^b^ |  |
| *PmPS5A* | *Xgwm526-2A* | CAATAGTTCTGTGAGAGCTGCG | CCAACCCAAATACACATTCTCA | 760,575,627 - 760,575,776 | (Niu et al., 2010) |
|  | *Xgwm382-2A^a^* | GTCAGATAACGCCGTCCAAT | CTACGTGCACCACCATTTTG | 772,967,310 - 772,967,422 |  |
| *Pm65* | *Xstars355* | AGAATGGGTGGGCCTATACC | GGGGAAAACGAGAGAGGAAC | 763,289,402 - 763,289,705 | (Li et al., 2019) |
|  | *Xstars356* | AAATACGAGAGGAGATAGTATGACG | ACTAGTGGGCTGTGCTGCTT | 763,821,128 - 763,821,516 |  |
| *PmX* | *Xgpw4456* | ATTAGTCTCCTCCTCCCTTTGG | AGTAGCCGGGGCAGAAATAG | 765,548,516 - 765,548,537^c^ | (Fu et al., 2013) |
|  | *Xpsp3039^a^* | GCATCCAAATCCCTAAACCG | AGCATGTGTGAGATAGACGG | 771,166,392 - 771,166,411^c^ |  |
| *Pm4b-Fed* | *JS717* × *JS718* | AGGTGGACATCCTAGGCGCT | GATCTGGGTACCACAGCACCG | - | (Sánchez-Martín et al., 2021) |
|  | *Pm4.1* | TGCATTCTGGACCCTGACTC | CACCATTGGAAGGATGAGCTG | - | (Yao et al., 2022) |
|  | *Pm4b-Fed-S* | ATGCGCAGTAACCTTGTCACT | TAACACGGAAGACATGGGCC | - | this study |
| ^a^ The closest flanking markers *Xhbg327* for *PmX* and *Xwmc317*/*Xwmc317* for *PmPS5A* cann't be detected on chromosome 2A; ^b^ Cann't be detected and no more flanking markers reported; ^c^ The position of reverse primer was not be detected. | | | | | |

Supplementary Table 2. *In silico* PCR analysis of selected powdery mildew resistance genes on chromosome 2AL

| **Gene** | **Flanking marker** | **Marker Size** | **Product size of in silico cloning ^a^** | **Reference** |
| --- | --- | --- | --- | --- |
| *PmM192145E8-9* | *Xsdauk13* | - | ND ^b^ | (Yu et al., 2018) |
|  | *Xsdauk682* | - | 247bp |  |
| *PmLK906* | *Xgdm93-2A* | 122+134+136 bp | 118bp | (Niu et al., 2008) |
|  | *Xgwm265* | 198+230+315bp | ND ^b^ |  |
| *PmPS5A* | *Xgwm526-2A* | - | 139bp | (Niu et al., 2010) |
|  | *Xgwm382-2A* | - | ND ^b^ |  |
| *Pm65* | *Xstars355* | 304bp | ND ^b^ | (Li et al., 2019) |
|  | *Xstars356* | 389bp | 381bp |  |
| *PmX* | *Xgpw4456* | - | ND ^b^ | (Fu et al., 2013) |
|  | *Xpsp3039^a^* | - | ND ^b^ |  |
| ^a^ Based on the *de novo* assembly of SN15218 DNA resequencing data; ^b^ Can’t be detected. | | | | |
